# Collectanea

**Published:** 1826-11

**Authors:** 


### COLLECTANEA.

Floriferus, ut apes, in saltibus omnia libant,
Omnia nos, itidem, depascimur aurea dicta.

### PATHOLOGY.

*Memoir on Putrid and Gangrenous Fever,* by A. L. I. Bayle.—At a time when a new system of Pathology, founded on solidism, rejects every fact which tends to prove the alteration of the animal fluids in certain diseases;—when it is attempted to reduce the immense series of maladies which afflict mankind, to one single primary lesion—*inflammation;*—when Therapeutics, founded on this idea, every where sees only a tendency to inflammation, and thus reduces almost all treatment to the employment of antiphlogistic remedies: at such a time it becomes the duty of every physician to pay undivided attention to clinical observation; to admit among his systematic and exclusive doctrines those only which are confirmed by facts; and to publish the cases which might destroy, confirm, or modify, preconceived opinions. This motive induces me to publish the two following cases; the first communicated to me by M. Blaynie, and the second by M. Breschet. I shall add some other facts of a similar nature, and some experiments on animals, which, although previously published, deserve to be brought together, and offered to the consideration of enlightened practitioners.

Case I. B——, aged eighteen, tall and slender, pupil at the Veterinary School of Alfort, came to consult me on the 13th of September, 1825, for a pain which had spontaneously come on the preceding night in the left ankle. There was swelling and a little redness round the articulation. Leeches were applied to the part, followed by emollient cataplasms, and quiet enjoined.

14th.—Redness and swelling increased. In the evening, slight shivering, followed by fever.

15th.—Pain more violent, and apparent fluctuation behind the outer ankle. On cutting deep in this situation, nearly an ounce of very thick pus escaped.

16th.—Violent fever, with headache, delirium, irregular motions of the limbs; breathing free; tongue clean, and not red.

17th.—Face pale, expression languid; eyes dull; delirium; no knowledge of his present condition, nor of those around him. Urine scanty; skin cold, covered with copious clammy sweat; pulse 110, small and sharp.

18th, (fifth day of the disease.)—Skin studded with an eruption of white pimples, as large as peas: one, situated on the external part of the left thigh, was as large as a bean, and quite black. For the first time, the patient refused to drink. During the day he lay on his back; great prostration; constant starting of the tendons; breathing oppressed. He died at four P.M.

Dr. Ramon and M. Girard, jun. Professor at Alfort, saw the patient several times the day he died, and being, like myself, unable to account for the kind of mischief which caused such severe and sudden symptoms, we agreed to open the body the next day.

*Appearances on dissection.*—The body smelt horribly, and would have been insupportable, but for the precaution of washing it with chlorate of lime. The skin was discoloured, and studded with pimples, having the form and size of peas, and which contained a whitish purulent matter: one of these, as already stated, was as large as a bean, and black. A deep incision in its centre showed that the skin, as well as the subjacent cellular tissue, participated in this gangrenous state. The wound, which was behind the outer ankle of the left limb, did not penetrate into the articulation; but the pus had diffused itself nearly a third of the length of the fibula, and had rendered the extremity carious.

In the *head*, the membranes and brain presented nothing remarkable, excepting that the middle lobe of the right hemisphere was a little softened.

*Thorax:* On opening the chest, there escaped a gangrenous, putrid, and insupportable smell. The lungs were evidently swollen, and entirely filled the two cavities of the chest: they were of a blackish hue, of a firm consistence, as if hepatised. On cutting into them, they exhaled a putrid smell, and a serous fluid exuded; they tore easily, and they were in such a state of disorganisation that neither the ramifications of the bronchiæ nor the cellular texture could be recognised. The portion of the pleura which formed the mediastinum was of a deep brown colour; and along the vertebral column, on the left side, were two longitudinal holes, one above the other, about an inch and a half each in diameter: that nearest the diaphragm corresponded to a similar opening on the right side. Their borders were black and ragged, and this portion of the pleura tore with great ease. These two ulcerations crossed the œsophagus. The muscular parietes of this canal were entirely destroyed for an inch and a half in extent, while the mucous membrane was untouched. This membrane, throughout the extent of the œsophagus, was of a deep brown colour; the chlorate of lime, which we were obliged to pour into the lungs, preventing our ascertaining the quantity and nature of the effusion, which no doubt had taken place. The heart was void of blood, but very large: its texture was so soft that the least violence tore it. The membrane lining the right auricle and ventricle was of a purplish red.

*Abdomen:* The liver and spleen were visibly enlarged, black and gangrenous, exhaling a putrid smell, which became stronger the deeper the incisions were made. Their texture had undergone such disorganisation, that their parenchyma was converted into a mass of putridity, easily reduced to rags by the touch of the fingers. The kidneys were sound. The digestive tube, seen externally, had a natural colour, and presented nothing particular from the rectum to the stomach.

This disease in its progress was so irregular and so rapid, that, until light was thrown upon it by the opening of the body, we thought that the greater part of the symptoms were caused by an affection purely cerebral. Having found, on dissection, a gangrenous disorganisation of the principal viscera of the chest and belly, without any premonitory symptoms of inflammation, we presume that such disorder could only proceed from the spontaneous development of a specific virus, which had caused the gangrenous disorganisation of the lungs, liver, spleen, and several places on the lateral part of the mediastinum and œsophagus.

Case II.—M. Girard, jun. Professor at Alfort,—complexion pale, and rather sallow,—had been long complaining of what he regarded as chronic inflammation of the digestive tube. He complained also frequently of pain and sanguineous congestions in his head. He had accustomed himself to apply leeches, or to be bled, on the least increase of his habitual state of suffering, which happened often more than once a month.

September 19th, 1825, M. Girard pricked the little finger of his left hand in assisting Dr. Blaynie in opening the body of the subject mentioned in Case I. Soon afterwards a little vesicle, surrounded with an inflammatory circle, appeared on the back of this finger, near its articulation with the metacarpal bone. M. G. irritated this little tumor in opening it with a pin, and afterwards with the point of a lancet. Some serum, at first reddish, then bloody, came from it. The whole limb became painful and swelled. M. Breschet was called in on the third day, and found the patient in a state of great suffering and anxiety. Leeches were applied, and next morning bleeding, during which he had a long-continued fainting fit. The finger was hard, swollen, œdematous, and emphysematous. There were the remains of a vesicle, the sides of which were red and inflamed. The fore-arm was swollen, and had several little inflammatory zones, similar to stains on paper. The affected part was cut into, and cauterised with nitras argenti; emollient cataplasms, local mucilaginous fomentations, and diluent drinks, were ordered.

The symptoms seemed to yield to this treatment. M. G. himself always dreaded the issue of the disease. The pains and inflammation of the arm and hand soon returned. Leeches were several times used, and calming narcotic fomentations and poultices sprinkled with laudanum; he had whey for drink; and, as he had much nervous irritability, they gave him, instead of opium, to which he had been habituated for some years, some grains of lactucarium, and a little of the extract of henbane. The inflammatory symptoms of the arm were never severe with regard to swelling and redness, but its sensibility was greatly increased, and the patient uttered loud cries on the slightest pressure. He complained at the same time of gastric and abdominal pains, and there appeared to be a connexion between these last and the state of the arm. The tongue was covered with a whitish coating, its edges slightly red, and the point of a colour a little more deep. The umbilical and epigastric regions were painful on pressure; stools scanty and mucous; skin clammy and moist; pulse frequent. The patient had great cerebral excitation without delirium, speaking rationally, receiving his friends, yet struck with a sense of the danger of his state, and of its fatal event. He was at this time seen by MM. Royer, Allard, Recumier, Husson, Dupuytren, and Bret. Warm baths were recommended, and gave relief. Leeches were several times applied to the abdomen, as well as poultices and emollient embrocations. Musk was given internally in the form of pills, and glysters.

It is worthy of remark that a pain came on at the articulation of the femur and tibia on the left side, and on the crest of the tibia. This at first resembled acute rheumatism, or deep-seated inflammation of the joint: the least pressure on the fore part of the limb caused the patient to cry out. There was no swelling or inflammatory redness; but two days after there appeared some little softness of the limb, in the point corresponding to the pain, and a careful examination gave a suspicion of pus under the tibial aponeurosis, although there was no evident fluctuation. The place was laid open, and there came forth about a spoonful of a greyish purulent matter, which seemed infiltrated in the cellular tissue between the muscles. The place was twice cauterised with nitrate of silver, and dressed so as to make the wound suppurate. The pain was lessened; no fresh inflammatory symptoms resulted from the cutting or caustic.

Towards the twelfth or fourteenth day, the disease assumed a more alarming aspect: the following symptoms came on:—Headache; intolerance of light; the greatest irritability; frequent irregular delirium; frequency, and sometimes irregularity of pulse; sleeplessness; agitation and subsultus of the tendons. These symptoms went on increasing, particularly the delirium. There was an abundant and very fetid diarrhœa. Baths, affusions, musk, and blisters to the legs, were tried. The patient at times recovered the use of his reason, but soon relapsed into his former state. Breathing became laborious, and the patient died after forty-eight hours of agony.

During the last two or three days, the body of the patient had a very fetid smell. About the middle of the course of the disease, there had been a slight and temporary eruption, and which had the appearance of measles.

The body was opened twenty-four hours after death.

*Head:* The arachnoid appeared moistened by a little more serosity than is usually observed: a layer of this fluid was infiltrated between it and the pia mater. The brain, cerebellum, cerebral protuberance, and spinal marrow, had a firmer consistence than usual, although the cerebral substance was very little injected. The ventricles contained an ounce of yellowish serum.

*Respiratory organs:* Each pleura contained six or eight ounces of a reddish-brown fluid, which appeared to be pure blood, or mixed with very little serosity: the right presented, at the upper part of the thorax, some old cellular adhesions. The lungs remarkably pale, a little greenish in the interior: their structure very crackling, but choked up with a great quantity of frothy serum. The larynx, trachea, and bronchiæ, natural.

*Circulatory organs:* These presented nothing remarkable.

*Digestive organs:* Periton[illegible]um pale and sound. Stomach contained some greyish liquid: its mucous membrane of a greyish yellow, and here and there the appearance of being injected; soft. The duodenum showed, throughout nearly all its extent, an emphysema under the mucous membrane; its inner coat raised in little phylyctenæ. Cells of the jejunum the same. The ileum more generally red, and especially towards the ileo-cœcal valve, where the redness was very deep. The large intestine contained gas; no fæces: its mucous membrane of a pale grey, except in some points, where there was a little injection. The colon in descending showed, a little above the sigmoid flexure, a narrowing, occupying three inches in length, and where its calibre was suddenly reduced to a third of that of the portions around it. This stricture appeared to be rather a fault of conformation than the result of disease: its sides, though a little thickened, were not hardened, showing no cicatrix, and was of a pale grey colour. The inner coat of the large intestine was generally thin. The structure of the liver was pale. The gall-bladder distended by thick and ropy bile, of a brown colour: its ducts were free. The pancreas stained with blood. The spleen of a colour like the grounds of wine. The kidneys, like the pancreas, were stained by a kind of ecchymosis; the ureters free. Bladder empty and sound; its mucous membrane white.

*Fluids:* The blood, except a small clot in the aorta, was fluid throughout; less abundant than usual, and showed a number of gaseous bubbles.

Case III.—A man, whose business was that of flaying dead animals, aged fifty, of a vigorous constitution, but an habitual drunkard, had been sick for six days. He lived in a high, dry, well-aired lodging, and was not in absolute poverty. He complained of great prostration of strength,—when lying on his back, he could scarcely raise his arms; tongue black and dry; breath fetid; look wild; eyes haggard; face pale, of a brownish-yellow colour, which contrasted with the striking redness of the right cheek; skin hot and pungent; urine thick, greyish yellow, and strong smelling; stools involuntary, liquid, bilious, and fetid; breathing slow and quiet; skin and flesh flaccid; pulse irregular, seventy-seven; delirium, but, when the patient's attention was fixed, he could answer questions put to him.

It was discovered that, on the night of August 2d, 1821, he came home drunk: the next morning he complained of headache and nausea, which he attributed to fatigue; however, he killed six horses, which he says had no contagious disease. On August 4th, after killing some horses, which he says were free from any disease, he occupied himself in stretching skins to dry: these skins had lain for several days heaped the one upon the other. In the evening he vomited. At ten o'clock he fainted twice, and was attacked with shivering and violent headache, which continued all night. The next morning he had strong fever, and on the left arm an itchy tumor, which he scratched till it bled. The fever and headache increased; he became delirious. Symptoms were a little easier in the evening, and increased in the night.

The third day, in the morning, slight epistaxis came on, without relief. A surgeon ordered twenty leeches to the temples, which took away a great deal of blood, and the patient again swooned twice. The blood was stopped by compression. The fever continued; the arm became excessively painful, the pain stretching up into the arm-pit; breathing hurried. He was bled. Next day, fever diminished towards evening, but increased in the night.

On the sixth day, the delirium was violent: this was removed by a second bleeding from the arm. and typhomania, and the symptoms before mentioned, came on. The arm was swollen from the shoulder down to the hand; the tumefaction soft, and feeling like emphysema. On the inner side, about two inches above the bend of the arm, there was a brownish-red shining depression, about the size of a shilling: ten or twelve phylyctenæ were scattered round this depression. We looked upon the disease as a malignant pustule, and not as erysipelas: in consequence, that part, which was really gangrenous, was scarified, and cauterised by introducing a piece of nitrate of silver. The blood which had been drawn from the arm was dissolved, black, and smelt like putrid flesh: it had only been drawn three hours and a half, when we carried it away, to make the experiments which will be mentioned. Wine, Decoction of Bark with Camphor, and Spiritus Mindereri, were prescribed. During the next night, two pustules appeared on the left thigh.

Seventh day, the state of the patient was very alarming. A potion, with Aqua Cannellæ, Æther, Spiritus Mindereri, and Vinum Cinchonæ. In the evening, the pustules on the thigh were gangrenous: they were cauterised with nitrate of silver.

Tenth day, the state of the patient was the same. In the morning there occurred a slight epistaxis of black dissolved blood. The smell of the patient was so offensive, that it was necessary to use nitric acid fumigations.

Eleventh day, in the morning, vomiting of yellow bile; bilious, liquid, and very fetid stools; clammy sweats on the trunk. Blisters applied to the limbs since the evening before had caused no redness. The tumor on the arm, on which compresses moistened with Vinum Cinchonæ were applied, was less œdematous, redder, but no trace of healthy inflammation appeared round the wide eschar that the caustic had left. The same on the thigh. Antiscorbutic wine, in the dose of a pound, was prescribed to be taken by spoonfuls during the twenty-four hours.

Thirteenth day, typhomania had ceased; pulse was full and regular; strength of the patient increased; healthy inflammation appeared round the eschars; the part of the skin on which blisters had been applied without effect, became red and inflamed. From this time the disease diminished, and, after twenty days of powerfully tonic and stimulating treatment, the patient was convalescent. The re-establishment of his health was rapidly accelerated by cold sponging of the body, with a decoction of sage and vinegar, at first warm, and then cold when his strength increased a little.

*General reflections.*—However numerous the points of difference in the preceding cases, one is struck, on reading them, with certain circumstances, which establish among them an identity of nature. They all tend to prove that there was in the subjects of them an alteration of the humours, and in some sort a putridity of them, which depended, in most cases, on external infection; and that this specific cause, at first concentrated on some limb, was afterwards absorbed, and affected a greater or lesser number of organs, causing various phenomena, which (if nature did not eliminate the poison by a critical effort) ended by giving place to a general contamination of the blood, and to a certain number of inflammatory, hemorrhagic, or gangrenous alterations of different parts. Thus, to prove the putridity and alteration of the blood, we see in the subject of the first case, by M. Blaynie, an exhalation towards the conclusion of an infected smell, and after death a general putridity. The patient of M. Breschet had offensive and putrid stools, and a smell of the same kind from his body, during the last days of the disease. The flayer, whose case is detailed, had the same. This fact is of the highest importance, as it evidently proves that the blood of the patient was already tainted with a certain degree of putrescence. Indeed, we have seen that the injection of blood taken from this patient into the veins of two animals, caused a mortal disease; and that the counter-proof, made on other animals with the blood of an ordinary patient, was followed by no unpleasant result.

The experiments of M. Gaspard confirm all these facts, in showing us the development of putrid diseases in animals into whose veins corrupted animal or vegetable matter has been injected. The same experiments prove also the efforts of nature to eliminate deleterious matters; as we see in the first cases, in which the substances injected had been less abundant, the re-establishment of health quickly followed the expulsion of frequent and very fetid stools. We may see also a tendency in nature to produce a crisis, (which was insufficient, it is true,) in the abscesses formed in different parts.

The tendency to gangrene is manifest in all the preceding cases. In the first, the gangrenous softening of the lungs of the liver and spleen, and two ulcerations of the posterior mediastinum and œsophagus; in the second, the small and fetid stools; in the third, phlyctenæ, and then black tubercles on the face; in the fourth, the infected smell, putrid dissolution of the blood, and brownish phlyctenæ on different parts. In fine, in the experiments of M. Gaspard and M. Gendrin, brownish and black spots on the lungs, &c.

What are the organs destroyed by the septic miasma circulating in the blood of subjects affected with putrid fever? Always the mucous membrane of the digestive organs, say certain physiologists. For ourselves, we go no further than the cases which are detailed above: thus, in the first, the gastro-pulmonary mucous membrane was only red, while the lungs, liver, and spleen, were disorganised by gangrene; in the second, the most important injuries existed in the membranes of the brain and the chest, the mucous membrane of the intestines being only injected; in the third, that membrane was sound. In fine, in the animals submitted to experiment, the intestines, it is true, were often affected, but other organs were always simultaneously diseased.

With regard to the treatment used in the cases above mentioned, when it is considered that it consisted almost entirely in the employment of antiphlogistics, and that the only patient who was cured began to mend when the most powerful tonic and stimulating plan was adopted,—we shall not be inclined to make it a rule to employ antiphlogistics in all diseases, and particularly not in putrid fever. If we consult authors, and compare numerous cases to be found in the works of Hoffman, Huxham, Leclerq de la Cloture, Pringle, Monro, Morton, &c. with those we have recited, we must be convinced that there is a striking analogy between them, and that they all owe their origin to one common cause,—namely, a septic alteration of the humours.

From the preceding cases, and from all those that we meet with on record, may be concluded—

1st. That the blood is susceptible of being primitively altered, and of undergoing a certain degree of putridity, either spontaneously or (which appears more frequently the case) under the influence of an external miasmatic infection.

2d. That such humoral depravation may occasion inflammatory or gangrenous affections of one or more organs, without its appearing to affect constantly the same.

3d. That gastro-enteritis is not constant in putrid fever, since, in the cases mentioned in this Memoir, it only showed itself once. (*Revue Medicale.*)

*Experiments on Animals, showing the result of Injecting various Putrid Substances.*—M. Gendrin made some experiments with the blood which had been lost by the subject of Case III. given above.

Experiment 1st.—One ounce of the blood, after being eight hours drawn, was injected into the cellular tissue of the groin of a cat. For an hour and a half, the animal was not in the least incommoded: he was offered drink, and took it with greediness. After an hour and fifty minutes, trembling of the lower lip, and soon after evident nausea. At the end of two hours, considerable vomiting of yellow bile, with great effort. Two and a half hours, very copious vomiting of greenish bile ; the animal laid itself on its side, and uttered plaintive cries on each inspiration, which appeared difficult and painful. The throat was half open, and the tongue between the teeth. The animal was set on his feet several times, and endeavoured to walk, but it soon stopped and laid itself down again. Six hours after the injection, the heat of the skin and ears was considerable; pulse small, frequent, and irregular; the tongue dry, and hanging out between the opened jaws. The animal died six hours and fifty minutes after the injection, with slight convulsive movements, in a state of complete prostration. We examined the body immediately. The skin in the groin was discoloured; the cellular tissue soft, pulpy, of a yellowish colour: it had a fetid smell, and was studded with little red marks. The mucous membrane of the stomach and intestines sound ; that of the trachea and bronchiæ, tinged of a reddish brown, was neither thickened nor inflamed. The lungs contained black blood, especially the left, and were studded with spots. The blood was liquid and black, even in the arteries. There were in the left pleura about two ounces of black and very serous blood. The heart was flaccid and soft, as if boiled, particularly the left side of it. No change in the brain or spinal marrow.

Experiment 2d.—Half an ounce of the blood lost by epistaxis was injected into the crural vein of a little dog: it had been five or six hours since it was lost by the patient; it had a heavy smell, not quite fetid; he agitated it strongly, to give it more liquidity. Four hours after the injection, the animal had nausea, and a vomiting of greenish bile: he was dejected; pulse frequent. Six hours, completely dejected; lying on its side, its mouth in the brownish stuff that it had vomited; the jaws closed. Seven hours after the injection, it died. On opening the body, one hour after death, it showed livid and black spots on the lungs, caused, as in the subject of the former experiment, by ecchymoses. The blood was black and liquid in all the vessels. The mucous membrane of the bronchiæ was brownish.

Experiment 3d.—M. GASPARD injected into the jugular vein of a dog about two ounces of white and fetid pus: soon after the animal fell into syncope, and vomited, which occurred six times in the course of the day. An hour after, an evacuation of excrement, with thick clouded urine, with slight relief. In the evening, its paws stretched out; breathing not to be discerned; pulse very weak. Ten hours after the experiment, this dog passed some black, liquid, extremely fetid stools, which gave relief, and the dog promptly recovered.

Experiment 4th.—The same experimentalist injected into the other jugular of the same dog nearly three drachms of the same pus; and at the end of a short time there came on, as in the first experiment, fainting, vomiting, frequent evacuation of urine; and, twelve hours after the injection, very watery, white, fetid stools; and, in twenty-four hours, death took place. On dissection, no remarkable change in the intestines or other organs.

Experiment 5th.—M. Gaspard repeated these two experiments on a pointer bitch, and attained each time similar results. After the first injection, the animal rallied; but it died after the second, made at an interval of two days. On dissection, no visible alteration, excepting that the inferior lobes of the lungs were inflamed, not crackling, almost hepatised, and sunk in water.—M. Gaspard several times repeated these experiments, and always with nearly the same results.

Experiment 6th.—He injected into the jugular of a little bitch half an ounce of fetid liquid, arising from the simultaneous putrefaction of beef and blood. In a moment afterwards, movements resembling the act of swallowing; soon after, dyspnœa, sinking, evacuation of excrement and urine. At the end of an hour, prostration of strength; gelatinous, bloody, and frequent stools; redness of the conjunctiva; belly tender to the touch; gradual diminution of strength; bilious vomiting; and death three hours after the injection. On opening the body, still warm, the lungs were found inflamed in a great degree, or rather gorged, not crepitating, of a violet or black colour, with many petechial blotches; which existed also in the tissue of the left ventricle of the heart, in the spleen, mesenteric glands, gall-bladder, and even in the subcutaneous cellular tissue. The mucous membrane of the stomach slightly red; that of the intestines, and chiefly of the duodenum and rectum, having a livid colour, with black points, covered with a gelatinous and bloody stuff, like the washings of flesh.

Experiment 7th.—M. Gaspard repeated the same experiment on a pretty large dog. Soon afterwards, evacuation of very fetid excrements, with much urine; frequent effort to expel fæces; breathing accelerated and deep; pulse small and frequent; sinking of strength. At the end of an hour, a kind of diarrhœa, marked by liquid, sanious, and fetid stools, which continued till death occurred, two hours and a half after the injection. On dissection of the body, yet warm, similar appearances were found as in the last experiment; the lungs studded with lucid, brownish-black spots, as large as a farthing; intestinal canal filled with mucous sanies, similar to that in the stools; mucous membrane of the intestines equally red, livid, and having an hemorrhagic inflammatory appearance. (*Ibid.*)

*Fœtus in Fœtu.*—In the last Number of Grafe and Walther's Journal, a case is related of a woman who was delivered of a dead male child, between the sixth and seventh month of pregnancy; at the posterior part of which, where the anus is usually situated, was appended a bag of skin. Upon examining its contents, which were almost in a state of putrefaction, they were found to consist of a placenta and a dead fœtus, apparently between the fourth and fifth month. The connexion between the fœtus and placenta was not to be clearly distinguished. It was not possible to make out all the parts of the former, in consequence of putrefaction. The head, brain, face, os sacrum, &c. lay in a confused mass of destruction. The bag of skin, which had supplied the place of an uterus, was not connected either with the pelvic or abdominal cavity, or with the spinal marrow, of the more perfectly formed fœtus. The rectum of the latter terminated in a cul-de-sac within the pelvis.

---

### PRACTICAL MEDICINE.

*On the Employment of Acetate of Morphia in Uterine Hemorrhagies;* by Dr. Fabre.—Mlle. L—, aged thirty-two, suffered during two years from hemorrhagy from the uterus every month, which considerably weakened a constitution originally vigorous, as evinced by her faded yellow complexion, and by continual pains in the hypogastric region and thighs, with frequent shiverings.—In the month of November, 1825, she sent for me, when I found her in bed, complaining of severe pain in her throat; the mouth was covered with aphthæ; tonsils swollen; the pharynx very painful; difficulty in swallowing, and sense of suffocation. She was at this time under her habitual monthly hemorrhage. Fifteen leeches were applied to the neck, and gave some relief: eighteen more were applied in the evening. Next day, the affection of the throat was greatly better, and the aphthæ soon disappeared. Low diet was continued, but still the hemorrhage remained the same; and the limbs became œdematous, even to the middle of the thighs. The syrup of the acetate of morphia was prescribed, in doses of three coffee-spoonfuls daily. Under this treatment the hemorrhagy diminished. The dose was gradually increased till the discharge ceased. She was soon able to go about again. This happened several times, the same measures each time being successful, until at last the menstrual discharge became regular; her complexion resumed a healthy look; pain was removed; the œdematous swelling disappeared; and she was perfectly cured.

Other cases are mentioned where the syrup of acetate of morphia was equally successful; and both M. Fabre and his colleague M. Ducros speak confidently of its virtues. (*Journal Complementaire.*)

---

### SURGERY.

*Operation of Tracheotomy for the Removal of a foreign Body in the Trachea.*—The following case is interesting, when taken in connexion with Dr. Webster's case given in the present Number. It is related by Dr. Atlee, iu the American Medical Review.

On Wednesday noon, August 11th, I was consulted by a son of Mr. Dinsmore, aged ten, who had that morning, while running, put a button-mole into his mouth, which, during respiration, was drawn into the trachea. He complained of uneasiness during respiration, attended with a very slight rattling, and pointed to the depression at the upper part of the sternum as the situation of the mole. He informed me that he had swallowed bread, and many other articles recommended to him, without effect. Upon requesting him to cough, a rattling was heard, and soon after a sudden check to expiration denoted the lodgment of the button against the lower surface of the glottis, which required a sudden and violent effort of inspiration to remove the sense of suffocation. Being an intelligent boy, I explained to him the nature of the case and the difficulty of dislodgment without an operation, and told him to inform his parents. I gave him an emetic, with the hope that the violent efforts at expiration produced by it might throw the mole through the rima glottidis. Next morning I was sent for by his mother, who told me that during the night he had two or three severe spells of coughing, which almost amounted to suffocation, with very great anxiety and alarm. These were relieved by placing him in an upright position, and keeping him so until morning. After explaining to her (the father being absent,) the nature of the accident,—the improbability of its removal through the natural passages,—the danger of delay producing great irritation, inflammation, and its consequences,—the safety, and almost certainty of success from an operation, both she and the boy expressed their willingness to submit to any mode of relief I thought proper to use.

Having given an opium pill about ten o'clock, to tranquillise the system, and ease the cough if possible, the patient was placed upon a dining-table, with his head bent over the edge, and held firmly by an assistant. An incision was made through the integuments, about an inch and a half long, extending downwards from above the cricoid cartilage. The sterno-hyoid and thyroid muscles were then separated and exposed, the inferior thyroid veins lying upon the trachea and passing down to the transverse vein. After exposing the trachea, a sharp-pointed bistoury was passed through it about the third ring, and extended downwards about three-quarters of an inch. From the symptoms previous to the operation, I concluded that the mole must lie upon the bifurcation of the trachea, its diameter (nearly half an inch) being too great to admit its entrance into the bronchiæ, and I depended more upon the expulsive power of the lungs than the use of forceps. Holding open the orifice of the wound, I requested him to cough: this he did several times violently, but in vain. Suspecting that it might have passed through the chink, and swallowed during the alarm of the operation, I shut up the trachea and the wound, and told him to cough again: the mole was immediately thrown up against the chordæ vocales, and he exclaimed, "It is there yet." Being satisfied as to its presence, I passed a probe through the wound as far as the bifurcation without feeling the mole, but the probe caused a violent effort to cough, when the probe was withdrawn, and immediately after it the mole was thrown through the wound several feet from me. The wound was closed by two interrupted sutures and adhesive straps, and the boy, relieved of all his uneasy sensations, was put to bed. (The boy did well.)

*On the Use of the Probe in any Operation in which a punctured Artery is to be tied.*—Connected with the very interesting case of wound of the carotid artery, in our last Number, we beg to introduce the following observations, from Mr. Hutchison's work on Surgery:—

When an artery is wounded by any sharp instrument, it will, I am sure, occur to every good surgeon that the best practice will be to tie the vessel above and below the punctured part, for reasons too obvious to notice; although it is not to be denied but that, in some situations, this may be a very difficult task. A task of such difficulty, we are informed, lately occurred in a large government hospital, in which the femoral artery was tied considerably above the punctured part, and which succeeded without any after-hemorrhage ensuing. That this method will not often so succeed, the knowledge we possess of the circulation by anastomosis too sufficiently proves, to authorise such a practice, when it is in our power to tie the vessel above and below the punctured part.

In cases where an artery is punctured by a sharp instrument, and it is our intention to tie it as recommended above, I cannot too strongly impress upon the minds of young surgeons the great facility with which the operation will be completed by the assistance of a common probe, introduced into the aperture of the vessel after the division of the external parts. The relation of the subjoined case will sufficiently illustrate the principle.

Thomas Mellifont, a seaman of the York (74), on the 16th of March, 1813, was attacked with incipient symptoms of pneumonia, for which the surgeon of the ship directed him to be bled. The assistant who performed the operation, owing to a pitch of the ship, then under sail, slit open the brachial artery more than a quarter of an inch in an oblique direction. The hemorrhage was suppressed by a thick compress and roller until the 17th, when the patient was placed under my care.

On the bandage and compress being removed, the blood gushed out in a saltus, and in larger volume than I supposed it possibly could have issued from the artery, if the vessel had been completely divided in a transverse direction. Pressure was therefore made upon the artery near the axilla, so as to stop the circulation of the vessel below, when I made an incision two inches in length over the puncture, and in the direction of the vessel, until, by gentle scratches with the scalpel, the punctured part of the artery was reached. Before we could clearly see the aperture made in the artery, however, three small branches were tied, which bled freely and obscured our view. A probe was now passed through the opening into the canal of the artery upwards, which enabled us to divide the vessel completely at this part, to raise the divided upper end into which the probe had been introduced, and to detach it a little way from the great radial nerve and its cellular connexions, until we could lay hold of it with a pair of artery forceps, by which it was drawn downwards, and the artery firmly tied with a ligature, in the same manner as in an amputated stump. In detaching the lower divided end of the artery with the probe introduced, a few fibres of the tendon of the biceps were necessarily divided, and a ligature was in like manner applied here.

The wound was cleared of blood, and the sides were brought together by slips of sticking plaster. The ligatures came away in a few days, the wound very soon cicatrised, and left the patient in as full possession of the use of his arm as if no such accident had occurred, and he was discharged to his duty.

In the year 1818, I performed the same operation, and for the same accident, on a patient of the Westminster General Dispensary. The operation in this case was conducted precisely in the same manner as that just described; and the great utility of the probe was here equally apparent. This man also recovered the perfect use of his arm in a very short time.

*Extirpation of the Testicle, in a Patient with a large Scrotal Rupture on the same side.*—Dr. Wedemeyer, of Hanover, lately removed the left testicle in a patient who had also, on the same side, a reducible scrotal rupture, of considerable magnitude. The only peculiarity in the operation was the care that was required to avoid injuring the hernial sac. It is worthy, however, of observation, that the rupture, which was reduced at the time of the operation, did not subsequently protrude. Considerable inflammation supervened after the operation, and it is presumed that the descent of the intestine was prevented by adhesions formed during its process, in the track through which the rupture had originally passed. (*Journal für Chirurgie*, band ix. stuck i.)

*Treatment of Syphilis without Mercury.*—A series of experiments, illustrative of the non-mercurial treatment of syphilis, have lately been made by Dr. Gunther, of Hamburg; and the results differ but little from those which have been derived by many surgeons of this country. Bleeding, low diet, and perfect rest, were the means principally employed. If the patients were anxious for a rapid cure, mercury was exhibited. In many cases it was found that buboes dispersed by pressure, even when matter could be distinctly felt in them. In females, chancres healed in from seven to twenty-one days; in men, they required from fourteen days to a month. (*Ibid.*)

